# Concerns regarding deployment of AI-based applications in dentistry – a review

**DOI:** 10.1038/s41405-025-00319-7

**Published:** 2025-03-25

**Authors:** Abhishek Lal, Ayesha Nooruddin, Fahad Umer

**Affiliations:** 1 Department of Medicine, Aga Khan University, Karachi, Pakistan; 2 Department of Surgery, Aga Khan University, Karachi, Pakistan

**Keywords:** Dentistry, Dental public health

## Abstract

**Introduction:**

Artificial Intelligence (AI) is a rapidly evolving technology, with various applications in dentistry including diagnosis, treatment planning, and prognosis. There are various AI-based applications for dental practitioners, however, their real-world evaluation through deployement studies is scarce, as most of the studies are validation studies. This review explores the potential pitfalls of focusing solely on technical performance metrics when evaluating AI-based applications in dentistry while overlooking the importance of clinical applicability.

**Methods:**

An electronic search was performed on PubMed and Scopus while a manual search was conducted on Google Scholar “Dentistry”, “Dental”, “Artificial Intelligence”, “Deep Learning, “Machine Learning”, “Applications”, “Diagnocat”, “CephX”, “Denti.AI”, “VideaAI”, “Smile Designer”, “Overjet”, “DentalXR.AI”, “Smilo.AI”, “Smile.AI”, “Pearl”, “AI deployment challenges in dental practice”, “AI for treatment planning in dentistry”, “AI in dental imaging”, and “AI-based diagnosis in dentistry”.

**Results:**

The electronic search yielded a total of 34 studies, while 10 additional studies were obtained through a manual search, resulting in a total of 44 studies included in this review.  Among the 44 studies analyzed, 26 studies were retrospective, while 7 studies utilized a comparative design. The remaining studies comprised of 3 observational, 5 validation, 2 cross-sectional, and 1 prospective study. Further to evaluate the identified applications, relevant companies were contacted via email. Only one company’s representative responded, offering a limited trial version which was insufficient for evaluating the application’s effectiveness. AI technologies may offer lots of benefits for dental practice by enhancing patient-health-based outcomes, however, real-world applications are necessary to ensure its safety.

**Conclusion:**

This work highlights the need for conducting deployment studies for such AI-based dental applications to translate and implement them into dental practice. Collaboration with stakeholders and dental practitioners to assess the use of such applications is of paramount importance.

## Introduction

Artificial Intelligence (AI) is emerging as a promising technology in dentistry, but its adoption in clinical practice is limited due to challenges such as the need for large training datasets, validation, data privacy, and deployment of AI-based applications. AI use is being proposed in terms of patient care including improved diagnoses, treatment planning, and prognosis [1]. AI technologies offer the potential to reduce human error, increase efficiency, and provide more precise, data-driven insights in dental care [2].

The dental literature is full of validation studies, which emphasize the vast array of small-scale investigations [3]. These studies predominantly focus on assessing an individual AI-based application tasked with specific objectives [4]. Within such research, the technical precision of these applications is extensively documented, utilizing a diverse set of robust performance metrics. These metrics include sensitivity, specificity, positive and negative predictive values, and the area under the receiver operating characteristic curve (AUROC) or precision-recall (PR) curve [5]. The metrics are crucial as they enable standardized reporting and facilitate comparison between applications. They should always serve as the cornerstone of any assessment of AI-based applications, however, relying solely on these metrics is insufficient. Performance metrics are critical for understanding the capabilities of AI-based applications, but they often fail to capture the broader clinical impact. For example, metrics like sensitivity and specificity provide insights into how well an AI tool can detect or rule out a condition, but they do not address how the tool will integrate into a clinical workflow or how it may affect patient outcomes.

Many practitioners hesitate to adopt AI-based applications in their clinical practice due to their concerns about reliability and potential errors [6]. Lack of transparency and information in terms of the deployment and assessment of AI-based applications are some of the key reasons for the same [7]. To ensure the acceptability and translation of AI into dental practice, there is a need to explore the real-world use of AI-based applications in dentistry.

Therefore, this review aims to explore the potential pitfalls of focusing solely on technical performance metrics when evaluating AI-based applications in dentistry. It also discusses the concerns related to the real-world deployment of these applications in dentistry, with an emphasis on their applicability and integration into clinical practice. The following case study illustrates a hypothetical scenario highlighting the importance of a comprehensive approach that prioritizes clinical impact of AI-driven dental care.

### Case presentation

A 12-year-old patient presented for a routine dental examination. The periapical radiograph, analyzed by the in-house AI software, flagged a suspected cavity on the lower first molar (tooth #36). Upon closer examination, the radiolucency appeared atypical, with a jagged outline and no surrounding radiolucency. Reviewing the patient’s dental history revealed a low caries risk and a recent sealant application on tooth #36. These findings suggested a mis-identification by the AI, likely due to an artifact from the fissure sealant.

In the presented case, focusing solely on the AI’s output could have led to unnecessary intervention due to automation bias [8]. The use of AI for caries detection has shown the potential of increased sensitivity, especially in early caries but with decreased specificity (False Positive) these errors are inevitable [9]. There is a need to convert errors or mis-identificationss made by AI applications into clear and informative messages that dentists can easily understand when making clinical decisions.

In this case, by considering the atypical radiographic presentation and the patient’s dental history, the dentist was able to correctly diagnose the finding as an artifact. Radiographic diagnosis serves as the foundation that guides clinicians, yet it alone does not determine a specific outcome. The clinical impact of diagnosing caries also depends on how the clinician responds to the radiograph. Several factors have to be considered such as caries risk, clinical exam, and disease prevalence before any treatment is undertaken.

**Fig. 1:**
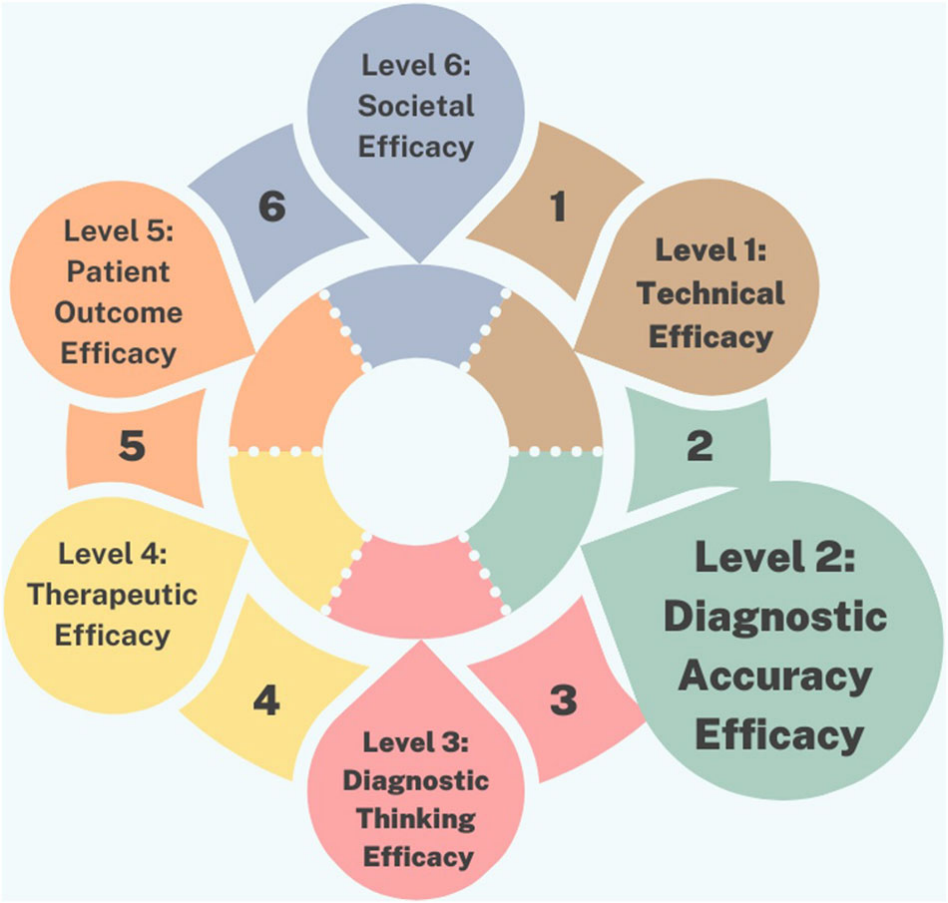
Levels of deployment of AI studies in dental care.

In essence, the efficacy of the diagnostic imaging process is multifaceted. Therefore Fryback and Thornbury suggested a comprehensive framework for evaluating its efficacy ranging from technical performance to societal impact [10]. This framework is illustrated in Fig. 1 and further explained in detail in the results section. Each level contributes to understanding the effectiveness of imaging technologies in improving patient care and informing healthcare policy decisions. This framework has been suggested for the assessment of the efficacy of AI-related applications in healthcare [11].

## Materials and Methods

### Research question

What are the concerns associated with the real-world deployment of AI-based applications, particularly regarding their integration into clinical practice and impact on patient outcomes?

### Search strategy

A search strategy defining the focus of interest such as dental applications that utilized AI models was developed by the authors. Initially, to evaluate the outcomes, the authors performed a preliminary search based on the specific keywords. The search strategy also included cross-referencing citations from key articles to identify additional relevant studies and expert opinions.

### Literature search

A manual Google search was performed initially to identify the most commonly utilized dental propriety applications which were then included in the search strategy to identify relevant studies. An electronic search was conducted using PubMed and Scopus with no restrictions on the year of publication and studies in the English Language to identify relevant literature. Furthermore, a manual search was performed using specific keywords on Google Scholar by the authors to identify studies that were not found in the electronic database search.

### Search terms

For a literature search, the following search terms were used: “Dentistry”, “Dental”, “Artificial Intelligence”, “Deep Learning, “Machine Learning”, “Applications”, “Diagnocat”, “CephX”, “Denti.AI”, “VideaAI”, “Smile Designer”, “Overjet”, “DentalXR.AI”, “Smilo.AI”, “Smile.AI”, “Pearl”, “AI deployment challenges in dental practice,” “ AI for treatment planning in dentistry,” “AI in dental imaging,” and “ AI-based diagnosis in dentistry”.

### Eligibility criteria

Inclusion criteria:

1. Original peer-reviewed studies
2. Studies related to dental AI-based applications
3. Studies that have been published to date

Exclusion criteria:

1. Studies such as all kinds of reviews including systematic reviews and meta-analysis, conference proceedings, case reports, case series, letter to the editors, editorials, and policy documents.
2. Studies published in languages other than the English Language

### Screening process

The initially identified studies were imported to Endnote version 21.0 (Clarivate Analytics) and the duplicates were removed. The title and abstract of each study were then screened as part of the initial screening by authors (AL and AN) analogously to the predetermined eligibility criteria. Any disagreement was resolved by discussion with the third author (FU). After initial screening, the remaining studies underwent full-text screening to determine the final count of the included studies in this review. The data extracted from the included studies was imported into pre-formulated proforma to chart the characteristics of included studies and key findings.

### Data extraction

A pre-formulated proforma was drafted using Google Sheets with the following variables: Author, year of publication, study objective, study design, name of AI-based application, key findings, limitation and level of deployment, services provided, availability of demonstration, approval from the Food and Drug Administration (FDA), and whether there are any peer-reviewed studies conducted on the respective AI-based application.

## Results

### Study selection and search results

The electronic search conducted through PubMed and Scopus identified a total of 776 studies out of which 51 duplicate records were removed. Out of the remaining 725 studies, 665 studies were excluded based on the irrelevancy of their titles. Following this, 60 studies underwent abstract and full-text screening, resulting in the inclusion of 34 studies from the electronic search. An additional manual search using Google Scholar yielded 123 studies, out of which 10 were included. In total, 44 studies were included in this review.

### Summary characteristics of studies

The studies included in this review were published between 2011 and 2024, with most of the studies reporting a sample size ranging from 10 to 68 patients. Among the 44 studies analyzed, 26 studies [12–37] were retrospective, while 7 studies [38–44] utilized a comparative design. The remaining studies comprised of 3 observational [45–47], 5 validation [48–52], 2 cross-sectional [53, 54], and 1 prospective study [55] (Supplementary Table). In terms of AI-based application utilization, Diagnocat was the most frequently employed tool, used in 26 studies [13–16, 18–24, 27, 28, 30, 32, 33, 35–37, 41, 43, 51, 52, 55], followed by CephX in 16 studies [12, 17, 25, 26, 29, 31, 34, 38, 40, 44, 45, 47–50, 54]. Additionally, 2 studies [39, 53] utilized Denti.AI, while Smile.AI [42] and Smile Designer [46] were each reported by 1 study. The deployment levels of the studies according to Fryback and Thornbury's model varied. Notably, 27 studies [13–16, 19, 21–23, 25, 26, 28, 30, 31, 33, 36, 39–41, 43–46, 50–53, 55] achieved level 2 deployment that assessed the AI software and tested their efficacy without any real-world deployment. However, 6 studies [20, 24, 38, 42, 47, 49] attained level 1, while another 6 studies [17, 18, 34, 35, 37, 48] reached level 3, and 3 studies [12, 29, 54] reached level 4. Only one study achieved level 5 [32] and level 6 each [27], respectively assessing the effectiveness of the AI-based application in clinical workflows. Regarding discipline, 20 studies had AI-based applications focusing in the field of Orthodontics [12, 17, 21, 25, 26, 29–31, 34, 36, 38, 40, 42, 44, 45, 47–50, 54], followed by 7 studies in Endodontics [13, 33, 35, 39, 41, 43, 52], 6 studies in Dental Radiology [14, 15, 22, 27, 32, 53], and 5 studies in Oral and Maxillofacial Surgery [16, 18, 23, 24, 28]. However, only two studies were in Prosthodontics [19, 46] and Periodontics [20, 51], and only 1 study for Restorative Dentistry [37] and Oral Medicine [55].

### Contacting companies

To assess the validity and deployment, we attempted to gather more information about AI-based applications for dentistry by reaching out to different companies via email claiming to offer AI solutions to dental practitioners, as presented in Table 1. The developers that we contacted included Smilo.AI ©, Smile.AI © Videa.AI ©, Diagnocat ©, Denti.AI ©, Smile Designer ©, Overjet ©, CephX ©, Pearl ©, and DentalXR.ai ©. Despite our multiple inquiries via email, only CephX company's developer replied offering a free trial instead to complete access which was needed to evaluate the real-world deployment of the application.

**Table 1:** Overview of AI-based applications in dentistry: services, regulatory status, and research support.

	Applications	Services	Demo Availability	FDA	Peer-Reviewed Papers
**1**	Diagnocat	Dental imaging (2D and 3D)	Available on request	NM	[27, 28, 30, 32, 33, 41, 43, 51, 52, 55]
**2**	Smilo.AI	Patient management	Available on request	NM	NM
**3**	Denti.AI	Dental X-ray imaging and voice charting	Available on request	Cleared	[39, 53]
**4**	Videa.AI	Dental imaging	Available on request	Cleared	NM
**5**	Smile Designer	Smile design based on different parameters	Available on request	NM	[46]
**6**	Overjet	Dental imaging (decay and bone loss detection)	Available on request	Cleared	NM
**7**	CephX	Cephalometric analysis and teeth segmentation	Available on request	NM	[12, 17, 25, 26, 29, 31, 34, 38, 40, 44, 45, 54]
**8**	DentalXR.ai	Dental X-ray imaging	Available on request	NM	NM
**9**	Smile.AI	Dental examination	Available on request	NM	[42]
**10**	Pearl	Dental X-ray imaging	Available on request	Cleared	NM

*NM* Not Mentioned, *FDA* Food and Drug Administration.

SI Table 1: Summary characteristics of included studies (*n* = 44).

Deployment levels refer to different stages of studies, ranging from technical reports (Level 1) to assessing the broader societal impact with levels 5–6 specifically denoting applications in real-world settings. The multi-level framework, as presented in Fig. 1, for evaluating AI in dental care is predominantly populated by studies focused on deployment Level 1: Technical Efficacy and Level 2: Diagnostic Accuracy Efficacy. These studies primarily involve principles of concepts, and validation that assess AI algorithm performance and diagnostic accuracy compared to expert human dentists. Level 1 studies emphasize parameters like algorithm accuracy, sensitivity to imaging artifacts, and computational efficiency, while Level 2 studies focus on metrics such as sensitivity, specificity, and positive predictive value. In contrast, there are comparatively fewer studies that explore the higher levels of effectiveness, such as Level 3: Diagnostic Thinking Efficacy, Level 4: Therapeutic Efficacy, Level 5: Patient Outcome Efficacy, and Level 6: Societal Efficacy.

## Discussion

### Fryback & Thornbury’s model in dental AI

Fryback and Thornbury’s hierarchical model has been widely used to evaluate the effectiveness of diagnostic technologies in medicine [10]. However, its application in dentistry has remained unexplored. To the best of the authors’ knowledge, this review is the first to adapt this framework to the dental context, providing a comprehensive evaluation of AI applications in dentistry.

According to this model, most studies included in this review reached levels 1 and 2, indicating that these were primarily validation studies which are conducted in controlled environments. Only two studies progressed to higher levels of the hierarchy, which evaluate AI’s impact on clinical decision-making, patient health outcomes, and societal benefits in real-world settings. Since validation studies do not account for the complexities and variability of real-world settings, the actual benefits and challenges these applications present for both providers and patients remain uncertain.

Notably, several AI-based dental applications, such as Pearl© and Overjet©, included in this review are currently well-known and widely available within the dental landscape. However, despite their popularity, there is a significant lack of studies evaluating their effectiveness. Therefore, it is essential that these applications undergo rigorous scientific evaluation to ensure their accuracy and reliability [56]. Additionally, the authors of this review contacted companies of identified AI-based applications but only one company responded. This leaves us with a significant gap in the transparency of AI-based applications in dental practices. It prompts a need for a closer examination to ensure that commercially available AI-based applications meet established standards for accuracy and dependibility. These applications should only be made accessible in the market after their effectiveness has been thoroughly tested, enabling dental professionals and patients to benefit from their use.

Almost all of the studies included in this review reported a limited sample size. This limitation highlights the challenges in generalizing the findings to broader populations and underscores the need for future research with larger, more diverse samples to ensure the reliability of these AI-based applications [57]. Additionally, there is considerable variation in the focus of AI-based applications across different dental disciplines. For instance, Orthodontics has emerged as the most extensively explored field in terms of using the AI based applications available, likely due to its high demand for precision in diagnosis and treatment planning. In contrast, fields such as Prosthodontics and Periodontics which also require precision remains underrepresented. Future research should focus on expanding the use of AI-based applications to these underexplored areas. This will ensure equitable technological advancements and patient care across all dental specialties.

### Regulatory clearance for AI-based applications in dentistry

AI-based dental applications, reported in this review, exhibit varying degrees of regulatory clearance. FDA clearance is an essential step in evaluating the safety, efficacy, and reliability of medical technologies, including AI-based applications [58]. For instance, Diagnocat© application which uses two-dimensional and Cone Beam Computed Tomography (CBCT) imaging to identify signs of cavities, periodontal diseases, and other pathologies is not yet FDA-cleared. This lack of clearance limits its use to research settings or regions where FDA oversight is not a requirement. Furthermore, the lack of FDA clearance also limits user’s confidence and trust in utilizing the application. In contrast, Overjet© has received FDA clearance for specific functionalities, such as evaluating the extent of periodontal bone loss and detecting decay on radiographs [59]. Similarly, another application in use called Denti.AI© is FDA -cleared which caters to transcribing patient-clinician conversations, improving diagnostic accuracy, perio charting, and identifying restorations through radiographs [60]. This clearance ensures that the application meets regulatory standards and has undergone rigorous evaluation for these purposes. However, FDA clearance alone does not guarantee optimal performance or successful clinical integration of these AI applications. Periodic updates and retraining are necessary to maintain their accuracy and relevance, especially as new data emerges, or clinical needs evolve [61].

### Recommended future metrics for AI in dentistry

The growing significance of AI in healthcare underscores the need for developing measures that precisely evaluate its effectiveness. For these measurements to adequately represent the real-world settings, they must transcend conventional metrics like accuracy and precision. Metrics that assess AI’s direct effects on patients are suggested, such as Patient-Centered Outcomes like morbidity or mortality rates, quality of life (QoL), and readmission rates [62]. Metrics related to equity are also crucial; these include bias identification, social effect evaluation, and disparity measures to guarantee that AI operates equally across demographic groups [62]. Important criteria in Clinical Decision Support (CDS), such as time efficiency, diagnostic accuracy, and adherence to recommendations, will be used to assess how well AI supports physicians [62]. Additionally, measurements for explainability, including Explainable AI (XAI) will guarantee that the rationale behind AI models is transparent and easy to comprehend. Lastly, Long-Term Impact measurements will evaluate AI’s potential to succeed in the changing healthcare environment. These indicators include sustainability, scalability, and adaptability. AI may be created and applied in ways that enhance patient care, healthcare outcomes, and equity by utilizing these thorough measures.

### Future of AI in dentistry

The burgeoning application of AI in dentistry necessitates the development of robust frameworks and guidelines which should clearly illustrate its development, testing, and deployment. This will ensure that AI applications are used safely and effectively in clinical settings, minimizing the risk of errors and maximizing patient benefits. Currently, AI systems in dentistry are often developed in isolation, using different methodologies, biased datasets, and algorithms, which can result in inconsistent outcomes across various applications [63]. Standardization of these factors would help ensure that AI-based dental applications corroborate consistency and reliability regardless of the specific tool being used. This will build trust among dental professionals, who will feel more confident adopting AI applications that have been rigorously tested and meet recognized benchmarks [64].

Regulatory bodies and professional dental associations should also work together with the developers and dental practitioners to ensure a smooth transition of AI-based dental applications into practice. These bodies should take an active role in establishing safety and efficacy standards for AI-based applications by creating clear guidelines that specify how these applications should be evaluated before they are approved for use in dental practices. Professional dental associations can play a key role by providing education and training to dentists on how to effectively use AI-based applications in their practice. Moreover, AI developers can consider the clinical realities of dental practice when designing and testing their applications by closely collaborating with dental practitioners. The developers should also be encouraged to offer trials of their applications to healthcare professionals so that they can test the application in their practices to provide valuable feedback. The stakeholders and policymakers should ensure these applications should undergo independent and rigorous assessments by expert healthcare professionals, data management, and security experts. This will ensure the accuracy, validity, and safety of such applications as they are directly involved in patient care.

## Conclusion

This study underscores the potential of AI in enhancing diagnostic accuracy and treatment planning in dentistry. However, it highlights a significant gap between the promise of AI technologies and their real-world integration. While FDA clearance and technical performance metrics are essential, they are insufficient to guarantee clinical applicability and successful adoption. To bridge this gap, rigorous testing, regular updates, and collaborative efforts among stakeholders, including developers, dental practitioners, and regulatory bodies, are crucial. Ensuring transparency, standardizing evaluation criteria, and prioritizing clinical relevance are also vital for effective AI integration into dental practice. Future research should focus on higher levels of deployment, evaluating AI’s societal and therapeutic efficacy to ensure its long-term success and trustworthiness in clinical settings.

## Supplementary information

SI Table 1

## Data Availability

Data sharing is not applicable to this article as no new data were created or analyzed in this study.
