# Supplementary material for: Concerns regarding deployment of AI-based applications in dentistry – a review
Source: BDJ Open. 2025 Mar 25;11:27. doi: 10.1038/s41405-025-00319-7 (PMC11937414; doi:10.1038/s41405-025-00319-7)
Supplement: Supplementary file 1 — SI Table 1 [file 41405_2025_319_MOESM1_ESM.docx]

| **Author and Year** | **Objectives** | **Study design** | **AI-based application** | **Key findings** | **Limitations** | **Level of deployment** | **Discipline** |
| --- | --- | --- | --- | --- | --- | --- | --- |
| Szuhanek et al. 2011 ^48^ | Evaluate cephalometric characteristics of Class III malocclusion patients using CephX | Validation | CephX | CephX provided reliable data for orthodontic diagnosis. | Small sample size and limited demographic diversity; reliance on static radiographic imaging. | 3 | Orthodontics |
| Bratu et al. 2014 ^45^ | Evaluate craniofacial morphology and dental changes in patients with Angle Class II division 2 malocclusion. | Observational | CephX | Increased posterior CB length, decreased gonial angle, and retroclined maxillary central incisors with obtuse interincisal angles. | Small sample size (25 patients) and lack of longitudinal data for growth prediction. | 2 | Orthodontics |
| Mosleh et al. 2016 ^12^ | Develop and evaluate Ceph-X, an automated 2D cephalometric analysis system. | Retrospective | CephX | Ceph-X demonstrated high accuracy, reduced processing time by 10x, and received excellent usability ratings. | Small sample size; excluded automatic landmark detection to minimize error. | 4 | Orthodontics |
| H.Alqahtani et al. 2020 ^38^ | Assess the reproducibility of linear and angular measurements of cephalogram tracings made with CephX & FACAD | Comparative | CephX | CephX and FACAD measurements were reproducible and comparable. | No inter-examiner variability, single-centre study. | 1 | Orthodontics |
| Naoumova et al. 2020 ^49^ | Compare accuracy and efficiency of CephX, CephNinja Dolphin and manual cephalometric tracings | Validation | CephX | CephX is fastest but less reliable | Variability in dental landmark identification; reliance on limited sample size and operator expertise. | 1 | Orthodontics |
| Orhan et al. 2020 ^13^ | Evaluate the diagnostic accuracy of a Diagnocat for detecting periapical pathosis on CBCT scans. | Retrospective | Diagnocat | Diagnocat showed high accuracy in detecting lesions and comparable volumetric measurements to manual segmentation. | Difficulty with large lesions, cortical bone perforations, and distinguishing soft tissues; variability in CBCT image quality. | 2 | Endodontics |
| Bayrakdar et al. 2021 ^14^ | Assess the performance of Diagnocat for dental implant planning using CBCT images. | Retrospective | Diagnocat | Diagnocat detected 95.3% of missing teeth regions and 72.2% of MCs | AI struggled with bone thickness measurement and sinus/fossa detection; small sample size. | 2 | Dental radiology |
| Ezhov et al. 2021 ^15^ | Evaluate the performance of Diagnocat for CBCT imaging diagnosis. | Retrospective | Diagnocat | Diagnocat showed improved diagnostic sensitivity and specificity compared to unaided groups, reduced diagnostic time | Limited variability in training datasets Excluded histopathological validation. Struggled with 4-root canals or periapical radiopacity. | 2 | Dental radiology |
| Orhan et al. 2021 ^16^ | Evaluate Diagnocat performance in detecting impacted third molars and their anatomical relationships using CBCT. | Retrospective | Diagnocat | High accuracy for impaction detection and anatomical relationships, moderate agreement for canal detection. | Lower accuracy for vertically positioned teeth; limited agreement for canal number detection. | 2 | Oral and Maxillofacial Surgery |
| Jeon et al. 2021 ^50^ | Compare AI-based and conventional cephalometric analyses. | Validation | CephX | AI achieves clinically acceptable accuracy | Small sample size, single AI software. | 2 | Orthodontics |
| Khalid et al. 2022 ^17^ | Compare CephX cephalometric analysis to manual tracing | Retrospective | CephX | CephX provided similar accuracy to manual tracings but lower agreement for FH and CB measurements. | Small sample size, inability to use reference planes with digital tracing, reliance on a single software. | 3 | Orthodontics |
| Bonfanti-Gris et al. 2022 ^53^ | Evaluate the diagnostic performance of Denti.AI in detecting and classifying dental structures and treatments on OPG | Cross-sectional | [Denti.AI](http://denti.ai/) | High accuracy (>80%) for metallic restorations, endodontic treatments, crowns, and implants; low accuracy (41.1%) for resin-based restorations. | Errors in classifying natural teeth vs. implant-supported crowns; low detection accuracy for resin-based restorations. | 2 | Dental radiology |
| Hamdan et al. 2022 ^39^ | Determine the efficacy of Denti.AI in assisting dentists with detecting apical radiolucencies on OPG. | Comparative | [Denti.AI](http://Denti.AI) | Denti.AI Significant improvement in detecting small lesions (14.5%) and lesions on endodontically treated teeth (15%). | Small sample size (68 cases). No analysis of false-positive rates in negative cases. | 2 | Endodontics |
| Orhan K et al. 2022 ^18^ | Develop and validate Diagnocat for pharyngeal airway detection in CBCT scans. | Retrospective | Diagnocat | Diagnocat's airway measurements aligned well with manual methods; | CBCT's low soft-tissue contrast affects segmentation; small sample size | 3 | Oral and Maxillofacial Surgery |
| Zadrożny Ł et al. 2022 ^19^ | Assess Diagnocat AI reliability in evaluating OPG | Retrospective | Diagnocat | Effective for identifying missing teeth and prosthetics but less reliable for caries and PLs. | Small sample size and reliance on manual evaluations as ground truth. | 2 | Prosthodontics |
| Al-Ubaydi et al. 2023 ^40^ | Evaluate the validity and reliability of tooth segmentation using CephX compared to intraoral scanning. | Comparative | CephX | High agreement between CephX and Insignia models (ICC > 0.88). CephX demonstrated high reliability for 3D tooth segmentation. | Small sample size (10 patients). Exclusion of cases with restorations and severe crowding | 2 | Orthodontics |
| Amasya et al. 2023 ^41^ | Evaluate the effect of using Diagnocat in CBCT for detecting dental caries. | Comparative | Diagnocat | Improved accuracy with AI: ROC 0.747–0.863 (unaided) to 0.903–0.920 (aided). | Artifacts and the absence of histopathological validation | 2 | Endodontics |
| Amasya et al. 2024 ^20^ | Develop and validate Diagnocat for periodontal bone loss detection using panoramic imaging. | Retrospective | Diagnocat | High performance: F-1 score: 0.996, accuracy: 0.993, and Kappa: 0.974 | Lack of standardization in imaging devices | 1 | Periodontics |
| Ali et al. 2023 ^42^ | Develop Smile.AI for digital smile design using ControlNet and segmentation models. | Comparative | [Smile.AI](http://smile.ai/) | High pixel accuracy with segmentation models (e.g., UNet++: 92% train, 73% test). | Limited dataset availability for before-and-after images. | 1 | Orthodontics |
| Balashova et al. 2023 ^21^ | Assess Diagnocat reliability in automatic detection of the upper airway on CBCT images. | Retrospective | Diagnocat | Reliability of 93.3% between AI and manual evaluations. | Small sample size (30 cases). | 2 | Orthodontics |
| Ceylan et al. 2023 ^46^ | Evaluate esthetic outcomes of digital smile designs generated manually or with Smile Designer AI. | Observational | Smile Designer | AI-generated designs were found acceptable for symmetrical faces | AI's effectiveness in cases with asymmetrical faces was questioned | 2 | Prosthodontics |
| Dirk Schulze et al. 2023 ^51^ | Compare the accuracy of Diagnocat and human observers in detecting periodontal lesions on CBCT datasets. | Validation | Diagnocat | Diagnocat underperformed compared to dentists, particularly for periradicular osteolysis and vertical bone loss. | Ineffective AI training and high variability in defect detection accuracy | 2 | Periodontics |
| Dadoush Maher et al. 2023 ^22^ | Compare the accuracy of Diagnocat and manual tracing software in detecting MCs on CBCT. | Retrospective | Diagnocat | Diagnocat achieved comparable accuracy to manual tracing, with no statistically significant differences in most regions of interest. | Challenges with metal artifacts, limited dataset size, and discontinuity in tracing MCs. | 2 | Dental Radiology |
| Issa et al. 2023 ^23^ | Evaluate the diagnostic accuracy of Diagnocat in detecting periapical periodontitis on PA | Retrospective | Diagnocat | Demonstrated high accuracy & reliability in identifying patterns and features unnoticeable to the human eye. | Small sample size (20 radiographs, 60 teeth). Data collected from a single source. Unequal distribution of healthy and unhealthy teeth in the dataset. | 2 | Oral and Maxillofacial Surgery |
| Issa et al. 2024 ^24^ | Compare the segmentation time and accuracy of AI-based inferior alveolar canal (IAC) segmentation with segmentation performed by a specialist. | Retrospective | Diagnocat | The AI-based segmentation closely matched the semi-automatic method in accuracy. Comparable segmentation times between the two methods. | Small sample size (15 CBCT scans, 30 IACs). Some areas of the IAC were incorrectly identified or omitted by the AI algorithm. | 1 | Oral and Maxillofacial Surgery |
| Kunz et al. 2023 ^25^ | Compare AI-based cephalometric analyses from four commercial providers to a human gold standard. | Retrospective | CephX | CephX showed significant deviations in 5/9 parameters. | Lack of diverse datasets; high variability in results for some parameters like incisor inclination | 2 | Orthodontics |
| Kazimierczak et al. 2023 ^26^ | Evaluate the correlation between nasal septum deviation (NSD) and AI-based cephalometric analysis. | Retrospective | CephX | Weak correlation between NSD and hinge axis angle (HAA); AI showed high repeatability in cephalometric analyses. | Small sample size, limited spatial analysis, exclusion of soft tissue analysis, and methodology differences. | 2 | Orthodontics |
| Orhan et al. 2023 ^27^ | Evaluate the reliability and accuracy of Diagnocat in identifying dental conditions using OPG | Retrospective | Diagnocat | High accuracy in diagnosing healthy teeth, crowns, implants, and periodontal bone loss; lower sensitivity for caries and PLs. | Single-center study with a limited dataset; poor reliability for certain pathologies like caries and pontics | 6 | Dental Radiology |
| Orhan et al. 2023 ^28^ | Assess the reliability of Diagnocat generated STL files in diagnosing osseous changes in mandibular condyles compared to expert radiologist. | Retrospective | Diagnocat | High reliability for flattening, bifid condyle, and osteophyte detection; poor reliability for osteosclerosis and early erosion detection. | STL files insufficient for trabecular bone or soft tissue evaluation | 2 | Oral and Maxillofacial Surgery |
| Paige A et al. 2023 ^29^ | Evaluate CephX accuracy for cephalometric analysis from CBCT scans compared to manual methods. | Retrospective | CephX | CephX showed acceptable error for 9/11 measurements. | Small sample, restricted patient diversity, requires more time and limited to the ABO analysis. | 4 | Orthodontics |
| Arslan et al. 2024 ^30^ | Evaluate AI performance in detecting and numbering teeth in cleft lip and palate patients. | Retrospective | Diagnocat | High overall sensitivity (0.98 ± 0.03) and precision (0.96 ± 0.04). | Limitations in cleft regions and challenges due to anatomical variations in left-sided clefts. | 2 | Orthodontics |
| Bor et al. 2024 ^31^ | Compare AI-assisted cephalometric analysis platforms with traditional digital tracing methods. | Retrospective | CephX | CephX showed more consistent results than manual tracing. | Potential errors in AI landmark detection without manual intervention; bias in datasets and calibration methods | 2 | Orthodontics |
| Boubaris et al. 2024 ^43^ | To compare Diagnocat with semi-automated segmentation for CBCTPAVI scores in PL volume assessment. | Comparative | Diagnocat | Diagnocat achieved accuracy: 91.3% (CBCTPAVI 1-2); F1-scores above 0.85 for indices 0, 3-6. | Inability to classify small lesions, multi-rooted lesions or open apex. | 2 | Endodontics |
| Gąbka K et al. 2024 ^32^ | Assess Diagnocat for identifying teeth in mixed dentition using OPG | Retrospective | Diagnocat | High accuracy for identifying third molars (100%). | Small sample size, restricted to OPG, and no CBCT validation. | 5 | Dental Radiology |
| Guinot-Barona et al. 2024 ^44^ | To compare cephalometric analyses performed by orthodontists with those performed by CephX | Comparative | CephX | CephX performed reliably for most landmarks | CephX struggled with complex ones like Xi or overlapping structures in incisor areas. | 2 | Orthodontics |
| Kazimierczak et al. 2024 ^47^ | Compare AI-driven and manual methods for facial asymmetry analysis using craniofacial CT scans. | Observational | CephX | AI lacked agreement with manual measurements; errors in 16.8% of cases; poor accuracy for asymmetry. | Small sample size, limited spatial analysis, exclusion of soft tissue analysis, and methodology differences. | 1 | Orthodontics |
| Kazimierczak et al., 2024 ^33^ | Evaluate the diagnostic accuracy of Diagnocat in assessing endodontic treatment outcomes using CBCT. | Retrospective | Diagnocat | High accuracy (>95%) for filling, obturation, density, and overfilling detection; moderate accuracy for voids (88.6%) | Small sample size, retrospective design, and variability in void detection accuracy | 2 | Endodontics |
| Kazimierczak et al. 2024 ^34^ | Evaluate the diagnostic accuracy of CephX in assessing RA and MC proximity. | Retrospective | CephX | AI showed high specificity but low sensitivity, performing best at a 0.5 mm threshold | Small sample size, use of CT instead of CBCT | 3 | Orthodontics |
| Kazimierczak W et al. 2024 ^52^ | Assess Diagnocat for endodontic treatment outcomes on OPG | Validation | Diagnocat | Accurate for filling but inconsistent for obturation and density | Small sample, single-center study | 2 | Endodontics |
| Kazimierczak W et al. 2024 ^35^ | Evaluate Diagnocat for detecting PL in OPG and CBCT images | Retrospective | Diagnocat | High specificity for both OPG and CBCT; better sensitivity with CBCT imaging | Small sample, single imaging device | 3 | Endodontics |
| Khabadze Z et al. 2024 ^36^ | Compare Diagnocat and manual methods for 3D cephalometric analysis | Retrospective | Diagnocat | Diagnocat performed well for mandibular growth and skeletal class but showed discrepancies in incisor and jaw angles | Small sample, limited parameters, and differences in landmark standardization | 2 | Orthodontics |
| Leśna et al. 2024 ^55^ | Assess patient fear and anxiety during dental hygiene visits using GBT and AI diagnostics | Prospective | Diagnocat | GBT and Diagnocat reduce anxiety and improve diagnostic precision | Small sample size; lack of diverse demographics; | 2 | Oral Medicine |
| Szabó et al. 2024 ^37^ | Assess the reliability of Diagnocat in diagnosing caries on intraoral radiographs. | Retrospective | Diagnocat | High specificity and accuracy; moderate sensitivity for caries detection. | Small sample size for bitewing radiographs; no clinical validation; variability in lesion depth assessments. | 3 | Restorative Dentistry |
| Zaheer R., et al. 2024 ^54^ | Compare accuracy and reliability of manual and CephX based cephalometric analysis. | Cross-sectional | CephX | CephX was most accurate and reproducible; manual methods had the highest detection errors. | Small sample size, focused only on lateral cephalograms, and excluded patients with craniofacial deformities. | 4 | Orthodontics |

**References (cited in the Supplementary Information section only)**

 12. Mosleh MA, Baba MS, Malek S, Almaktari RA. Ceph-X: development and evaluation of 2D cephalometric system. BMC Bioinforma. 2016;17:499. https://doi.org/10.1186/s12859-016-1370-5.

13. Orhan K, Bayrakdar IS, Ezhov M, Kravtsov A, Özyürek T. Evaluation of artificial intelligence for detecting periapical pathosis on cone-beam computed tomography scans. Int Endod J. 2020;53:680–9. https://doi.org/10.1111/iej.13265.

14. Kurt Bayrakdar S, Orhan K, Bayrakdar IS, Bilgir E, Ezhov M, Gusarev M, et al. A deep learning approach for dental implant planning in cone-beam computed tomography images. BMC Med Imaging. 2021;21:86. https://doi.org/10.1186/s12880-021-00618-z.

15. Ezhov M, Gusarev M, Golitsyna M, Yates JM, Kushnerev E, Tamimi D, et al. Clinically applicable artificial intelligence system for dental diagnosis with CBCT. Sci Rep. 2021;11:15006. https://doi.org/10.1038/s41598-021-94093-9.

16. Orhan K, Bilgir E, Bayrakdar IS, Ezhov M, Gusarev M, Shumilov E. Evaluation of artificial intelligence for detecting impacted third molars on cone-beam computed tomography scans. J Stomatol, Oral Maxillofac Surg. 2021;122:3337. https://doi.org/10.1016/j.jormas.2020.12.006.

17. Khalid RF, Azeez SM. Comparison of cephalometric measurements of on-screen images by CephX software and hard-copy printouts by conventional manual tracing. J Hunan Univ Nat Sci. 2022;49:294–302.

18. Orhan K, Shamshiev M, Ezhov M, Plaksin A, Kurbanova A, Ünsal G, et al. AI-based automatic segmentation of craniomaxillofacial anatomy from CBCT scans for automatic detection of pharyngeal airway evaluations in OSA patients. Sci Rep. 2022;12:11863. https://doi.org/10.1038/s41598-022-15920-1.

19. Zadrożny Ł, Regulski P, Brus-Sawczuk K, Czajkowska M, Parkanyi L, Ganz S, et al. Artificial intelligence application in assessment of panoramic radiographs. Diagnostics. 2022;12:224.

20. Amasya H, Jaju PP, Ezhov M, Gusarev M, Atakan C, Sanders A, et al. Development and validation of an artificial intelligence software for periodontal bone loss in panoramic imaging. Int J Imaging Syst Technol. 2024;34:e22973. https://doi.org/10.1002/ima.22973.

21. Balashova M, et al. Artificial intelligence application in assessment of upper airway on cone-beam computed tomography scans. J Int Dent Med Res. 2023;16:105–10.

22. Dadoush, M. *Pre-implant tracing of mandibular canals on CBCT images using artificial intelligence*. University of British Columbia, (2023).

23. Issa J, Jaber M, Rifai I, Mozdziak P, Kempisty B, Dyszkiewicz-Konwińska M. Diagnostic test accuracy of artificial intelligence in detecting periapical periodontitis on two-dimensional radiographs: a retrospective study and literature review. Medicina. 2023;59:768.

24. Issa J, Kulczyk T, Rychlik M, Czajka-Jakubowska A, Olszewski R, Dyszkiewicz-Konwińska M. Artificial intelligence versus semi-automatic segmentation of the inferior alveolar canal on cone-beam computed tomography scans: a pilot study. Dent Med Probl. 2024;61:893–9.

25. Kunz F, Stellzig-Eisenhauer A, Widmaier LM, Zeman F & Boldt J. Assessment of the quality of different commercial providers using artificial intelligence for automated cephalometric analysis compared to human orthodontic experts. Journal of Orofacial Orthopedics/Fortschritte der Kieferorthopädie, 2023. https://doi.org/10.1007/s00056-023-00491-1.

26. Kazimierczak N, Kazimierczak W, Serafin Z, Nowicki P, Lemanowicz A, Nadolska K, et al. Correlation analysis of nasal septum deviation and results of AI-driven automated 3D cephalometric analysis. J Clin Med. 2023;12:6621.

27. Orhan K, Aktuna Belgin C, Manulis D, Golitsyna M, Bayrak S, Aksoy S, et al. Determining the reliability of diagnosis and treatment using artificial intelligence software with panoramic radiographs. Imaging Sci Dent. 2023;53:199208. https://doi.org/10.5624/isd.20230109.

28. Orhan K, Sanders A, Ünsal G, Ezhov M, Mısırlı M, Gusarev M et al. Assessing the reliability of CBCT-based AI-generated STL files in diagnosing osseous changes of the mandibular condyle: a comparative study with ground truth diagnosis. Dentomaxillofacial Radiology 2023;52:20230141. https://doi.org/10.1259/dmfr.20230141.

29. Paige A. *Comparison of Conventional and Automated Cephalometric Analysis Using Cone-Beam Computed Tomography*. (University of California, Los Angeles, 2023).

30. Arslan C, Yucel NO, Kahya K, Sunal Akturk E, Germec Cakan D. Artificial intelligence for tooth detection in cleft lip and palate patients. Diagnostics. 2024;14:2849.

31. Bor S, Ciğerim SÇ, Kotan S. Comparison of AI-assisted cephalometric analysis and orthodontist-performed digital tracing analysis. Prog Orthod. 2024;25:41. https://doi.org/10.1186/s40510-024-00539-x.

32. Futyma-Gąbka K, Piskórz M, Smala K, Miazek W, Moskwa M, Różyło-Kalinowska I. Evaluation of effectiveness of a virtual AI-based dental assistant in recognizing mixed dentition on panoramic radiographs. J Stomatol. 2024;77:1815. https://doi.org/10.5114/jos.2024.143587.

33. Kazimierczak W, Kazimierczak N, Issa J, Wajer R, Wajer A, Kalka S, et al. Endodontic treatment outcomes in cone beam computed tomography images—assessment of the diagnostic accuracy of AI. J Clin Med. 2024;13:4116.

34. Kazimierczak W, Kazimierczak N, Kędziora K, Szcześniak M, Serafin Z. Reliability of the AI-assisted assessment of the proximity of the root apices to mandibular canal. J Clin Med. 2024;13:3605.

35. Kazimierczak W, Wajer R, Wajer A, Kiian V, Kloska A, Kazimierczak N, et al. Periapical lesions in panoramic radiography and CBCT imaging—assessment of AI’s diagnostic accuracy. J Clin Med. 2024;13:2709.

36. Khabadze Z, Mordanov O, Shilyaeva E. Comparative analysis of 3D cephalometry provided with artificial intelligence and manual tracing. Diagnostics. 2024;14:2524.

37. Szabó V, Szabó BT, Orhan K, Veres DS, Manulis D, Ezhov M, et al. Validation of artificial intelligence application for dental caries diagnosis on intraoral bitewing and periapical radiographs. J Dent. 2024;147:105105.

38. Alqahtani H. Evaluation of an online website-based platform for cephalometric analysis. J Stomatol Oral Maxillofac Surg. 2020;121:53–57.

39. Hamdan MH, Tuzova L, Mol A, Tawil PZ, Tuzoff D, Tyndall DA. The effect of a deep-learning tool on dentists’ performances in detecting apical radiolucencies on periapical radiographs. Dentomaxillofacial Radio. 2022;51:20220122. https://doi.org/10.1259/dmfr.20220122.

40. Al-Ubaydi AS, Al-Groosh D. The validity and reliability of automatic tooth segmentation generated using artificial intelligence. Sci World J. 2023;2023:5933003. https://doi.org/10.1155/2023/5933003.

41. Amasya H, Alkhader M, Serindere G, Futyma-Gąbka K, Aktuna Belgin C, Gusarev M et al. Evaluation of a decision support system developed with deep learning approach for detecting dental caries with cone-beam computed tomography imaging. Diagnostics (Basel) 2023;13. https://doi.org/10.3390/diagnostics13223471.

42. Ali, GAK. et al. in *2023 Eleventh International Conference on Intelligent Computing and Information Systems (ICICIS)*. 568–73 (2023).

43. Boubaris M, Cameron A, Manakil J, George R. Artificial intelligence vs. semi-automated segmentation for assessment of dental periapical lesion volume index score: a cone-beam CT study. Computers Biol Med. 2024;175:108527.

44. Guinot-Barona C, Alonso Pérez-Barquero J, Galán López L, Barmak AB, Att W, Kois JC, et al. Cephalometric analysis performance discrepancy between orthodontists and an artificial intelligence model using lateral cephalometric radiographs. J Esthet Restor Dent. 2024;36:555–65.

45. Bratu DC, Bălan RA, Szuhanek CA, Pop SI, Bratu EA, Popa G. Craniofacial morphology in patients with Angle Class II division 2 malocclusion. Rom J Morphol Embryol. 2014;55:909–13.

46. Ceylan G, Özel GS, Memişoglu G, Emir F, Şen S. Evaluating the facial esthetic outcomes of digital smile designs generated by artificial intelligence and dental professionals. Appl Sci. 2023;13:9001.

47. Kazimierczak N, Kazimierczak W, Serafin Z, Nowicki P, Jankowski T, Jankowska A, et al. Skeletal facial asymmetry: reliability of manual and artificial intelligence-driven analysis. Dentomaxillofacial Radio. 2024;53:52–59.

48. Szuhanek C, Gâdea Paraschivescu E, Sişu AM, Motoc A. Cephalometric investigation of Class III dentoalveolar malocclusion. Rom J Morphol Embryol. 2011;52:1343–6.

49. Meriç P, Naoumova J. Web-based fully automated cephalometric analysis: comparisons between app-aided, computerized, and manual tracings. Turkish J Orthod. 2020;33:142–149.

50. Jeon S, Lee KC. Comparison of cephalometric measurements between conventional and automatic cephalometric analysis using convolutional neural network. Prog Orthod. 2021;22:1–8.

51. Schulze D, Häußermann L, Ripper J, Sottong T. Comparison between observer-based and AI-based reading of CBCT datasets: an interrater-reliability study. Saudi Dent J. 2024;36:291–5.

52. Kazimierczak W, Wajer R, Wajer A, Kalka K, Kazimierczak N, Serafin Z. Evaluating the diagnostic accuracy of an AI-driven platform for assessing endodontic treatment outcomes using panoramic radiographs: a preliminary study. J Clin Med. 2024;13:3401.

53. Bonfanti-Gris M, Garcia-Cañas A, Alonso-Calvo R, Salido Rodriguez-Manzaneque MP, Pradies Ramiro G. Evaluation of an Artificial Intelligence web-based software to detect and classify dental structures and treatments in panoramic radiographs. J Dent. 2022;126:104301.

54. Zaheer R, Shafique HZ, Khalid Z, Shahid R, Jan A, Zahoor T, et al. Comparison of semi and fully automated artificial intelligence driven softwares and manual system for cephalometric analysis. BMC Med Inform Decis Mak. 2024;24:271. https://doi.org/10.1186/s12911-024-02664-3.

55. Leśna M, Górna K, Kwiatek J. Managing fear and anxiety in patients undergoing dental hygiene visits with guided biofilm therapy: a conceptual model. Appl Sci. 2024;14:8159.
